# Cardiac remodeling in elite young Asian female soccer players

**DOI:** 10.3389/fcvm.2024.1404780

**Published:** 2024-11-15

**Authors:** Narae Kim, Hyunkyung Park, Il Suk Sohn, In-Ho Yang

**Affiliations:** ^1^ Department of Cardiology, Kyung Hee University College of Medicine, Kyung Hee University Hospital at Gangdong, Seoul, Republic of Korea; ^2^ Department of Emergency Medicine, Seoul Medical Center, Seoul, Republic of Korea

**Keywords:** athlete's heart, echocardiography, soccer, sports cardiology, cardiac remodeling

## Abstract

**Introduction:**

An athlete's heart (AH) varies depending on race, sex, age, and the type and intensity of training. Given that soccer is a common sport, evaluation of cardiac function in soccer players is important; however, few studies have analyzed adolescent soccer players. Therefore, this study, aimed to identify early changes in AH in soccer players by evaluating the echocardiographic findings of adolescent Asian female soccer players for whom existing data are scarce.

**Methods:**

We evaluated 20 Korean female under-20 national soccer team players and 42 age-matched healthy females. Participants provided physical examination data such as height, weight, blood pressure, and pulse, along with echocardiographic results. On echocardiography, parameters related to the morphology of the left atrium (LA) and left ventricle (LV) and those related to the systolic and diastolic function of the LV were measured.

**Results:**

The athlete group had a lower blood pressure and pulse rate than the control group. Echocardiography indicated that both the LA volume index and LV volume in the athlete group were large by approximately 1.5 times compared with those in the control group. The LV wall thickness and mass index were also significantly large in the athlete group. For LV diastolic function, parameters indicating early diastolic filling were substantially higher in the athlete group than in the control group.

**Conclusions:**

Adolescent female soccer players exhibited differences in cardiac morphology and an enhanced diastolic function. Therefore, this study suggests that AH begins in adolescence, with changes in both morphology and function.

## 1 Introduction

The term athlete's heart (AH) refers to the state in which the heart undergoes adaptations in response to continuous high-intensity training (1, 2). Continuous training results in an increase in the chamber size and wall thickness (2). Regarding cardiac function, the left ventricular (LV) stroke volume increases, whereas LV systolic function is normal or slightly decreased in response to high-intensity training (2). AH induces structural and functional changes, and its manifestation varies depending on factors such as race, sex, age, and type and intensity of training (1, 3).

Soccer is one of the most common sports globally. As the number of soccer players increases, the number of cardiac arrests occurring during soccer games increases (1). Accordingly, the need for cardiac evaluation in soccer players is increasing. The Fédération Internationale de Football Association (FIFA) requires cardiac function evaluation, including electrocardiography and echocardiography, before international matches (1). In a recent study, elite American soccer players demonstrated several echocardiographic parameters that exceeded the normal range outlined in existing guidelines (1). However, recent studies have shown significant differences in structural and functional cardiac remodeling in male and female athletes (4). Although female athletes are known to tend to have eccentric LV hypertrophy, reports of AH in females were rare (4). Differentiating between AH and pathological changes in athletes is important because pathological diseases that exhibit a morphology similar to AH can cause sudden cardiac death (3).

We analyzed the echocardiograms of Korean female under-20 (U-20) national soccer team players to determine differences occurring in the hearts of young Asian female athletes. Young athletes are likely to display differences similar to those in adults; however, limited data are available on young athletes and early changes in AH remain unknown (3). Furthermore, data on Asian populations are lacking because most studies on AH have been conducted in Caucasian populations (5, 6). According to studies conducted in Singapore and Malaysia, cardiac remodeling in Asian athletes demonstrated similar patterns, such as large chamber size and wall thickness; however, these changes were not as severe as those in Caucasian athletes (5, 6). A recent study of Taiwanese military women showed that structural and functional differences in the heart are not characteristic of Asian female athletes (7). Notably, both studies were conducted on adults, and no study has investigated adolescent Asian athletes. Therefore, this study aimed to (1) investigate the pattern of AH in Korean adolescent female soccer players, (2) identify any differences from their non-athlete peers, and (3) determine the early changes in the heart caused by training in Asians.

## 2 Methods

### 2.1 Design and participants

This cross-sectional observational study compared the heart structure and function between Korean female U-20 national soccer team players and healthy young adolescents. National soccer team players aged 17–20 years underwent echocardiography as part of their medical examination in July 2022 to participate in a FIFA match. The control group consisted of age-matched healthy females (aged 17–20 years) who underwent echocardiography at Kyung Hee University Hospital at Gangdong between October 2018 and August 2022. Individuals with severe systemic disease, those who did not consent to the use of their personal information, those who had previously undergone heart-related procedures or surgeries, those with underlying cardiac dysfunction, or those with congenital heart disease were excluded. Physical examination and echocardiography results were obtained for all participants. The physical examination parameters included height, weight, body mass index (BMI), body surface area (BSA), blood pressure (BP), and heart rate (HR). BSA was calculated using the Du Bois and Du Bois formula: Body Surface Area = 0.007184 × [Height(cm)^0.725^] × [Weight(kg)^0.425^]. We analyzed the differences in cardiac structure and function by comparing clinical characteristics and echocardiographic data between the two groups. The requirement for informed consent was waived because of the retrospective nature of data collection. This study was approved by the hospital's Ethics Committee (KHNMC 2023-04-010-001).

### 2.2 Echocardiographic examination

Echocardiography was performed with the patient in left decubitus position. Left atrium (LA) size, LV geometry and function, and ascending aorta diameter were evaluated in all participants. LA size was assessed by measuring LA diameter and volume index using M-mode and two-dimensional (2D) echocardiography. LV geometry was also evaluated using M-mode and 2D echocardiography. The LV internal dimensions at end-diastole (LVIDd), LV end-diastolic volume (LVEDV), and LV end-systolic volume (LVESV) were measured to evaluate the LV size. LV wall thickness was evaluated by measuring intraventricular septal thickness (IVSd), LV posterior wall thickness (LVPWd), LV mass index, and relative wall thickness (RWT). LV mass index was calculated using the following formula: LV mass index = LV mass/BSA, LV mass = 0.8{1.04[([LVEDD + IVSd + PWd]^3^ − LVEDD^3^)]} + 0.6. The linear dimensions of the LA and LV were measured in the parasternal long-axis view, and the volumes of the LA and LV were measured in a biplane in the apical view. LV systolic function was assessed by measuring LV ejection fraction (EF) using the biplane Simpson's technique. LV diastolic function was evaluated using early diastolic mitral inflow (E) velocity, late diastolic mitral inflow (A) velocity, septal early diastolic mitral annular (e’) velocity, lateral e’ velocity, E/A ratio, and E/e’ ratio. Pulsed-wave Doppler images of the mitral valve tip were acquired to measure peak E velocity, peak A velocity, and E/A ratio. Septal and lateral e’ velocities were measured in the basal inferoseptal and basal anterolateral walls, respectively, using tissue Doppler imaging. The E/e’ ratio was determined as the ratio of peak E velocity and peak septal e’ velocity.

### 2.3 Statistical analysis

Statistical analysis was performed with SPSS ver 13.0. Continuous variables are expressed as mean values and standard deviations. For each variable, the differences between the athlete and control groups were compared using an independent *t*-test. *p* < 0.05 was considered statistically significant. Power analysis was performed using PASS 16.0 software to evaluate whether the sample size was sufficient.

## 3 Results

### 3.1 Study population

A total of 62 adolescents aged 17–20 years were enrolled in this study. Twenty were Korean female U-20 national soccer team players and 42 were healthy teenagers. The athletes had less than 10 years of athletic experience, with 7–10 years of experience. Although the amount of training in healthy teenagers has not been investigated, athletes, engaged in 3–4 h of training per day. The average age of participants was 19 years (Table 1). Height, weight, and BSA were higher in the athletes than in the healthy adolescents; however, no significant differences in BMI were observed (Table 1). Both BP and HR were lower in the athletic group than in the control group (Table 1).

**Table 1:** Clinical characteristics of the study population.

Variables	Athletes (*n* = 20)	Non-athletes (*n* = 42)	*p*-value	Cohen's *d*
Age, years	19.2 ± 1.0	19.0 ± 0.80	0.533	0.22
Height, cm	167.4 ± 6.3	162.2 ± 4.4	<0.001	0.96
Weight, kg	61.1 ± 7.3	54.7 ± 11.7	0.010	0.66
BMI, kg/m^2^	21.7 ± 1.5	20.8 ± 4.4	0.219	0.27
BSA, m^2^	1.69 ± 0.12	1.57 ± 0.14	0.002	0.92
sBP, mmHg	108.2 ± 9.1	116.2 ± 19.6	0.032	0.52
dBP, mmHg	62.1 ± 6.4	68.4 ± 10.9	0.006	0.70
HR, bpm	57.8 ± 7.1	77.5 ± 15.2	<0.001	1.66

BMI, body mass index; BSA, body surface area; sBP, systolic blood pressure; dBP, diastolic blood pressure; HR, heart rate.

### 3.2 Echocardiography

Echocardiographic data are presented in Table 2. Significant differences were observed in the echocardiographic parameters between the two groups. These data showed a sufficient power of over 85% for these parameters (Supplementary Table S1). In terms of overall size, the structures were larger and the diameter of the ascending aorta was dilated in the athlete group.

**Table 2:** Echocardiographic parameters in athletes and non-athletes.

Variables	Athletes (*n* = 20)	Non-athletes (*n* = 42)	*p*-value	Cohen's *d*
LA diameter, cm	3.5 ± 0.3	3.0 ± 0.4	<0.001	1.41
LA volume index, ml/m^2^	29.4 ± 4.0	19.4 ± 4.5	<0.001	2.35
IVSd, cm	0.8 ± 0.1	0.7 ± 0.1	0.010	1.00
LVIDd, cm	4.9 ± 0.4	4.3 ± 0.4	<0.001	1.50
LVPWd, cm	0.8 ± 0.1	0.7 ± 0.1	0.016	1.00
LV mass index, g/m^2^	76.1 ± 12.1	58.8 ± 15.6	<0.001	1.24
RWT	0.3 ± 0.1	0.3 ± 0.1	0.827	0
LVEDV, ml	104.6 ± 12.2	69.8 ± 15.7	<0.001	2.48
LVESV, ml	36.8 ± 5.2	24.7 ± 6.1	<0.001	2.13
LV EF,%	64.8 ± 2.3	64.5 ± 4.5	0.669	0.084
E velocity, cm/sec	95.2 ± 16.4	88.3 ± 13.4	0.084	0.46
A velocity, cm/sec	37.3 ± 9.0	50.8 ± 11.6	<0.001	1.30
E/A ratio	2.7 ± 0.8	1.8 ± 0.4	<0.001	1.42
Septal e’ velocity, cm/sec	14.5 ± 1.9	12.8 ± 2.0	0.024	0.87
Lateral e’ velocity, cm/sec	18.8 ± 2.3	16.5 ± 2.5	0.001	0.96
E/e’ ratio	6.8 ± 1.1	7.0 ± 1.2	0.637	0.17
Aorta diameter, cm	2.7 ± 0.2	2.5 ± 0.2	<0.001	1.00

LA, left atrium; IVSd, intraventricular septal thickness; LVIDd, left ventricle internal dimension at end-diastole; LVPWd, left ventricle posterior wall thickness; RWT, relative wall thickness; LVEDV, left ventricle end-diastolic volume; LVESV, left ventricle end-systolic volume; EF, ejection fraction; E, early diastolic mitral inflow; A, late diastolic mitral inflow; e’, early diastolic mitral annular.

Regarding the structure of the heart, the diameter of LA was large in the athlete group (3.5 ± 0.3 vs. 3.0 ± 0.4 cm; *p* < 0.001). Considering the LA volume index, which corrects for the effect of BSA between groups, the size of the LA in the athlete group was approximately 1.5 times more dilated than that in the control group (29.4 ± 4.0 vs. 19.4 ± 4.5 ml/m^2^; *p* < 0.001). All parameters measuring LV size were greater in the athletic group than in the control group. LVEDV and LVESV were approximately 1.5 times larger in the athletes than in the healthy controls (104.6 ± 12.2 vs. 69.8 ± 15.7 ml; *p* < 0.001; 36.8 ± 5.2 vs. 24.7 ± 6.1 ml; *p* < 0.001).

The LV wall was thicker in the athletic group than in the control group. IVSd, LVPWd, and LV mass index were also large in the athlete group (0.8 ± 0.1 vs. 0.7 ± 0.1 cm; *p* = 0.010; 0.8 ± 0.1 vs. 0.7 ± 0.1 cm; *p* = 0.016; 76.1 ± 12.1 vs. 58.8 ± 15.6 g/m^2^; *p* < 0.001). However, RWT was within the normal range with no significant difference between the two groups (0.3 ± 0.1 vs. 0.3 ± 0.1; *p* = 0.827).

Regarding cardiac function, the average EF in both groups was around 65%, demonstrating no significant difference between the two groups (64.8 ± 2.3 vs. 64.5 ± 4.5%; *p* = 0.669). In terms of diastolic function, no difference was noted in the peak E velocity between the two groups (95.2 ± 16.4 vs. 88.3 ± 13.4 cm/sec; *p* = 0.084). However, peak A velocity was lower in the athlete group than that in control group (37.3 ± 9.0 vs. 50.8 ± 11.6 cm/sec; *p* < 0.001), which resulted in the E/A ratio being higher in the athlete group (2.7 ± 0.8 vs. 1.8 ± 0.4; *p* < 0.001). Peak septal e’ and lateral e’ velocities were also higher in the athlete group (14.5 ± 1.9 vs. 12.8 ± 2.0 cm/sec; *p* = 0.024; 18.8 ± 2.3 vs. 16.5 ± 2.5 cm/sec; *p* = 0.001). The result produced a slightly low E/e’ ratio in the athlete group, but the difference was not significant (6.8 ± 1.1 vs. 7.0 ± 1.2; *p* = 0.637).

## 4 Discussion

This is the first study to compare the echocardiographic findings between Korean adolescent female soccer players and healthy individuals. Many previous studies have analyzed AH in adult Caucasians; however, data on Asian, female, and young athletes are limited (5–8). Therefore, the strength of this study can be attributed to the rarity of participant groups. We aimed to analyze AH in a homogenous patient group of adolescent Asian female soccer players and compare the results with existing results. The main findings of this study are as follows: First, BP and HR were lower in the athletes than in the healthy controls. Second, the left heart and ascending aorta were dilated in the athletic group. Third, in the athlete group, the LV wall was thicker, but the RWT was within the normal range, resulting in a normal LV geometry. Fourth, the systolic function of the LV was normal, and for the LV diastolic function, the parameters indicating early diastolic filling were greater in the athletic group.

Asians have a smaller BSA than Caucasians; consequently, the degree of cardiac remodeling appears to be less pronounced in Asians compared to that in other racial groups (7, 9–12). Female athletes have smaller absolute cardiac dimensions than male athletes (9, 13, 14). As individuals age and training continuously, chamber size is gradually increases (9, 15). Based on previous studies, the changes in the chamber size and wall thickness in the athlete group in this study were expected to be relatively small.

However, the LA and LV chamber sizes in the athlete group were larger than those in the control group. The LA volume index in the athlete group was 1.5 times more than that in healthy participants, and the LVEDV in the athlete group also displayed a dilatation of 1.5 times. Although the participants included in previous Asian studies were female athletes in their 20s, their LVEDV was lower than that observed in this study (5, 6). This disparity was likely attributable to previous studies that analyzed athletes from various sports. Previous studies seem to have reported less chamber dilatation than this study because a significant number of power- and skill-type athletes were included. Traditionally, it has been known that endurance training causes changes in eccentric LV hypertrophy patterns, whereas resistance training causes concentric LV hypertrophy (9, 16, 17). However, recently published studies presented conflicting results with this hypothesis (17–19). Recent studies demonstrated that morphologic changes in the LV occur with endurance training, while resistance training does not cause changes in cardiac morphology (17, 18). Endurance training also appears to be related to both volume and pressure load on the heart (18). AH is associated with the time of training load, and it is skeptical that the AH phenotype depends on the type of training (18, 19). Supporting these findings, cardiac remodeling was noticeable in this study with more endurance-trained athletes. A previous study showing significant LV dilatation in female elite polo athletes and mild changes in female elite fitness athletes supported these findings (20). In a study analyzing adult soccer players in the United States, LV and LA chamber sizes were dilatated prominently, as observed in this study (1). This suggests that LA and LV dilatation occur in soccer players during early training loading.

LV wall thickness, as assessed by IVSd and LVPWd, got thicker in the athlete group. The LV mass index adjusted for BSA also were high in the athlete group. According to a previous study, data from adult female soccer players in the United States demonstrated that the LV mass index was an average of 91 g/cm^2^ (1). The results of this study revealed that, in the case of adolescent Asian athletes, the parameters were relatively small owing to differences in age and race. However, no significant difference in the RWT was observed between the athlete and control groups. In a study that serially observed adolescent cross-country skiers, after concentric remodeling occurred in the early stages of training, chamber dilatation occurred and RWT returned to normal or decreased (21).

In a study of elite judo athletes, LV dilatation was more prominent than LV wall hypertrophy (22). Similar outcomes were presumed in soccer. In this study, the thickness of the LV wall was high, but the RWT was not different from that of healthy individuals owing to the dilatation of the chamber. The athlete group showed a slightly eccentric LV pattern, but did not reach the point of eccentric hypertrophy, resulting in normal LV geometry, as in the control group. This result is consistent with recent studies demonstrating that most female athletes competing at the Olympics display normal LV geometry (23). This also matches with a recent study which showed that although the athlete cohort had higher LV mass index and wall thickness compared to the healthy cohort, the median value was in the normal range, resulting in normal LV geometry (24).

Systolic function did not differ between the athletes and healthy individuals. In previous studies, depending on the type of training, the EF of athletes was the same or slightly lower than that of healthy individuals (9). Some studies reported low EF in endurance-type athletes (9, 25). This is attributable to the reduced contraction force required to achieve the stroke volume when the LV is dilated (9, 23). This is the result of certain endurance-type athletes with eccentric LV hypertrophy (9, 26). Most athletes, including soccer players, exhibit LV systolic function similar to that of healthy people (1, 9, 23). The athletes in this study demonstrated normal LV geometry and systolic function, similar to those in the control group.

LV diastolic function in the athlete group was normal. Peak E velocity was similar between the athlete and control groups, and peak A velocity was lower in the athlete group, indicating an elevation in the E/A ratio in the athlete group. Previous studies presented that the mitral E/A ratio was higher in athletes than in healthy (27). Most studies have demonstrated normal or enhanced LV diastolic function in athletes (28, 29). As training load is continuously applied, early diastolic filling is enhanced, resulting in a decrease in the A velocity and an increase in the E/A ratio in athletes (5, 9, 29–31). According to a previous study, septal and lateral e’ velocities increased and the E/e’ ratio decreased because early relaxation of the LV increased (1, 5, 9, 29–31). Although the E/e’ ratio in this study was not significantly low in the athlete group, the septal and lateral e’ peak velocities were elevated. This indicates that the diastolic function was enhanced in the athlete group, as suggested in previous studies. As the training load continues, the change in e’ velocity, which measures myocardial velocity, is expected to become significant. A recent study analyzing adult female athletes in Asian demonstrated that the septal and lateral e’ peak velocities were higher than those in this study and the E/e’ ratio of athletes was significantly low (5). In a study analyzing adult soccer players in the United States, lateral e’ peak velocity was higher than that in this study (1). However, results regarding diastolic function based on the type and intensity of training are controversial; therefore, further studies are required (9, 32, 33).

Although the results of this study are similar to those of some previous studies, the strength and significance of our findings are as follows: First, compared to a previous study analyzing soccer players, although the athletes in this study had a small dilatation in chamber size and LV mass index, the patterns were similar. This implies that even adolescent Asian female soccer players exhibit significant differences in cardiac morphology. Second, compared to studies analyzing Asian adult athletes, the athletes in this study had greater dilatation of the LV chamber, indicating that the type and intensity of training have a significant impact on AH. Finally, in this study, the differences in the parameters measuring diastolic function were relatively small but notable. The enhancement of diastolic function appears to be at the beginning of the training.

### 4.1 Limitation

This study has several limitations. First, the sample size was small and there are limitations in generalizing the results because the study used data from national youth teams in a single country. However, power analysis showed that the sample size was sufficient. This suggests that significant differences existed between the groups. Second, since this was a retrospective study, the data that could be obtained were limited. Detailed echocardiographic parameters of the right heart were not obtained in most control group participants, and strain analysis was not performed. As a result, additional differences that exist between athletes and healthy individuals may not have been evaluated. Third, as this was a cross-sectional observational study, it had several limitations, including difficulty in identifying causal relationships. Multiple variables, such as diet, family history, and socioeconomic status, may affected the results. Moreover, elite athletes may have innate and genetic qualities that distinguish them from healthy people. These qualities can affect structural and functional differences in the heart. Although age, race, and sex were matched in this study, bias may have occurred because of these uninvestigated variables. To supplement the limitations of a cross-sectional study, a follow-up study with a larger sample size is required in the future.

## 5 Conclusion

Compared with healthy adolescents, young soccer players with <10 years of athletic experience demonstrated differences in cardiac morphology, such as chamber dilatation and enhanced diastolic function. Therefore, we can assume that in female soccer players, remodeling of cardiac morphology and function occurs at the beginning of training. We expect that additional evidence will be obtained in a longitudinal study with a larger sample size.

## Data Availability

The raw data supporting the conclusions of this article will be made available by the authors, without undue reservation.
