# Supplementary material for: Cardiac remodeling in elite young Asian female soccer players
Source: Front Cardiovasc Med. 2024 Nov 15;11:1404780. doi: 10.3389/fcvm.2024.1404780 (PMC11604807; doi:10.3389/fcvm.2024.1404780)
Supplement: Supplementary file 1 [file Table1.docx]

| **Variables** | **Athletes**  **(n = 20)** | **Non-athletes**  **(n = 42)** | **power** |
| --- | --- | --- | --- |
|  | **μ ± σ** | **μ ± σ** |  |
| LA diameter, cm | 3.5 ± 0.3 | 3.0 ± 0.4 | 0.9997 |
| LA volume index, mL/m^2^ | 29.4 ± 4.0 | 19.4 ± 4.5 | 1.0000 |
| IVSd, cm | 0.8 ± 0.1 | 0.7 ± 0.1 | 0.9479 |
| LVIDd, cm | 4.9 ± 0.4 | 4.3 ± 0.4 | 0.9997 |
| LVPWd, cm | 0.8 ± 0.1 | 0.7 ± 0.1 | 0.9479 |
| LV mass index, g/m^2^ | 76.1 ± 12.1 | 58.8 ± 15.6 | 0.9967 |
| RWT | 0.3 ± 0.1 | 0.3 ± 0.1 | 0.0500 |
| LVEDV, mL | 104.6 ± 12.2 | 69.8 ± 15.7 | 1.0000 |
| LVESV, mL | 36.8 ± 5.2 | 24.7 ± 6.1 | 1.0000 |
| LV EF, % | 64.8 ± 2.3 | 64.5 ± 4.5 | 0.0635 |
| E velocity, cm/sec | 95.2 ± 16.4 | 88.3 ± 13.4 | 0.3556 |
| A velocity, cm/sec | 37.3 ± 9.0 | 50.8 ± 11.6 | 0.9984 |
| E/A ratio | 2.7 ± 0.8 | 1.8 ± 0.4 | 0.9953 |
| Septal e’ velocity, cm/sec | 14.5 ± 1.9 | 12.8 ± 2.0 | 0.8844 |
| Lateral e’ velocity, cm/sec | 18.8 ± 2.3 | 16.5 ± 2.5 | 0.9372 |
| E/e’ ratio | 6.8 ± 1.1 | 7.0 ± 1.2 | 0.0972 |
| Aorta diameter, cm | 2.7 ± 0.2 | 2.5 ± 0.2 | 0.9479 |

**Supplementary Table. Power analysis of echocardiographic parameters**

LA, left atrium; IVSd, intraventricular septal thickness; LVIDd, left ventricle internal dimension at end-diastole; LVPWd, left ventricle posterior wall thickness; RWT, relative wall thickness; LVEDV, left ventricle end-diastolic volume; LVESV, left ventricle end-systolic volume; EF, ejection fraction; E, early diastolic mitral inflow; A, late diastolic mitral inflow; e', early diastolic mitral annular
